# Analysis of Difference in Microbial Community and Physicochemical Indices Between Surface and Central Parts of Chinese Special-Flavor *Baijiu Daqu*

**DOI:** 10.3389/fmicb.2020.592421

**Published:** 2021-01-14

**Authors:** Yanru Chen, Kaimin Li, Ting Liu, Ruyi Li, Guiming Fu, Yin Wan, Fuping Zheng

**Affiliations:** ^1^Beijing Advanced Innovation Center for Food Nutrition and Human Health, Beijing Technology and Business University, Beijing, China; ^2^State Key Laboratory of Food Science and Technology, School of Food Science and Technology, Nanchang University, Nanchang, China; ^3^Beijing Laboratory of Food Quality and Safety, School of Light Industry, Beijing Technology and Business University, Beijing, China

**Keywords:** special-flavor *Baijiu*, *Daqu*, microbial community, physicochemical indices, Illumina MiSeq sequencing

## Abstract

Special-flavor *Baijiu* is a unique *Baijiu* in Jiangxi Province, China, whose uniqueness mainly depends on the unique production process of special-flavor *Baijiu Daqu*. However, the microbial structure and physicochemical indices of different parts of the special-flavor *Baijiu Daqu* are still unknown. This greatly reduces the actual value of *Daqu* in the production of special-flavor *Baijiu*. Therefore, culture-dependent and Illumina MiSeq sequencing methods were used to analyze the microbial structure of special-flavor *Baijiu Daqu*. The results indicated that there was a complicated microbial diversity in Chinese special-flavor *Baijiu Daqu*. The predominant bacterial communities were Bacillales, Lactobacillales, and Rhodospirillales, while Saccharomycetales and Eurotiales were the predominant fungal communities. Significant differences in microbial community and distribution were shown between the surface and central parts of *Daqu*. *Acetobacter* and *Pichia* genera were the predominant microorganisms in the surface part of *Daqu*, whereas *Aspergillus*, *Kroppenstedtia*, *Oceanobacillus*, and *Bacillus* genera were the predominant microorganisms in the central part of *Daqu*. Meantime, the different microbial distributions between the surface and central parts of *Daqu* caused the significant differences in the physicochemical indices. These results can provide an important theoretical basis for improving the brewing process and the quality of special-flavor *Baijiu*.

## Introduction

Chinese *Baijiu* is an old distilled alcoholic beverage, which plays a crucial role in Chinese tradition, culture, and people’s daily diet. *Daqu* is a crude fermentation starter, which serves as one of the carriers of microorganisms during the fermentation process of Chinese *Baijiu* (Zheng et al., 2011). The production of Chinese *Daqu* adopts a natural inoculation process, and its microbial community comes from raw materials and the air, wherein a complicated microbial community suitable for the specific environment in *Daqu* gradually forms (Quigley et al., 2012). The main microbial communities of *Daqu* can be divided into bacteria, mold, and yeast. “Saccharification sees mold, yeast to ferment, raw fragrance by bacteria” is an interesting statement that indicates the role of *Daqu* microorganisms during the fermentation of Chinese *Baijiu*. Studies have proved that the aroma profile of Chinese *Baijiu* is the result of the metabolic activity of its microbial community (Li et al., 2017). Therefore, *Daqu* is one of the key impact factors that determine the quality and flavor of Chinese *Baijiu*.

The traditional method is to use the whole *Daqu* for Chinese *Baijiu* fermentation, and its use is more dependent on human experience (Zheng et al., 2012). However, with the development of detection methods, the changes in physicochemical indices, enzyme systems, and microbial communities of different parts of *Daqu* gradually become the new standard for *Daqu*’s application in Chinese *Baijiu* fermentation (Yang Y. et al., 2019). It has been reported that there was a significant difference in microbial community and distribution between the surface and central parts of *Jiang*-flavor *Baijiu Daqu*, and there was a significant difference in volatile compounds between the surface and the center of *Daqu* (Jin et al., 2019). Yang Y. et al. (2019) found that different microbial communities could influence the activity of different fermentation-related enzymes between the surface and central parts of medium- and high-temperature *Daqu*, such as esterase, saccharifying enzyme, and acid protease. These enzymes have an important influence on the formation of liquor flavor. Therefore, it is important to study the physicochemical indices and microbial communities between the different parts of *Daqu* to promote the scientific development of the modern Chinese *Baijiu* fermentation industry.

Special-flavor *Baijiu*, which is one of 12 types of flavored *Baijiu* in China, has a distinctive aroma with a high concentration of odd-numbered fatty acid ethyl acetate as the primary flavor compound, which contributes to the harmonious and strong flavor (Zheng and Han, 2016). The geographical and climatic conditions in the middle reaches of Ganjiang River in the Jiangxi Province of China provide a suitable environment for the natural inoculation of microorganisms in the special-flavor *Daqu*. For medium- to high-temperature *Daqu*, its manufacturing technique adopts an open tempering process that involves natural inoculation. In general, Chinese traditional *Daqu* is made from a mixture of barley and wheat. However, the production process of special-flavor *Daqu* is unique because it uses vinasse from pits as one of the raw materials and selectively inoculates acid-tolerant microorganisms in the natural environment (Li et al., 2020). However, the microbial communities and physicochemical indices in different parts of special-flavor *Daqu* are still unknown.

Therefore, the objectives of this study were to analyze the microbial structure of special-flavor *Baijiu Daqu* and to evaluate the potential effects between the microbial structure and physicochemical indices of different parts of *Daqu* by using culture-dependent and Illumina MiSeq sequencing methods. It is of great significance to guide the production of *Daqu* and improve the quality of special-flavor *Baijiu*.

## Materials and Methods

### Sample Collection and Pretreatment

**FIGURE 1:**
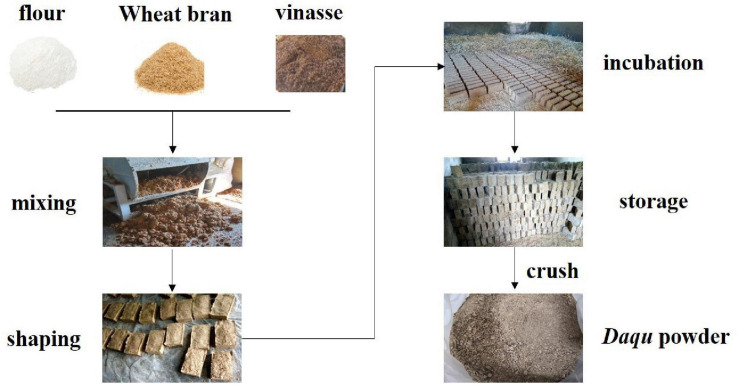
Schema of traditional method of making special-flavor *Baijiu Daqu.*

Special-flavor *Daqu* was obtained from the Jiangxi Zhangshugong Wine and Spirits Co. Ltd (longitude 115.53° and latitude 28.07°). Special-flavor *Daqu* was made from a mixture of 50% flour, 40% wheat bran, and 10% vinasse, which were mixed with warm water after grinding. The *Daqu* brick (27 cm × 17 cm × 8 cm) was constructed from the mixture and spontaneously incubated and fermented in a *Qu* room, which is a special open room near the Ganjiang River. Special-flavor *Daqu* belongs to medium- to high-temperature *Daqu*, whose fermentation temperature is between 40 and 50°C. The total fermentation period of special-flavor *Daqu* generally takes approximately 30 days through solid-state fermentation, and the dimension of mature *Daqu* blocks is 24 cm × 14.5 cm × 7.5 cm (Figure 1).

Mature special-flavor *Daqu* samples (after 30 days of fermentation) were collected according to the description of Shi et al. (2009). We randomly selected three mature *Daqu* bricks from the room where *Daqu* is stored. Each *Daqu* block was divided into two parts: the surface layer of 1.5 cm thick was named the surface part of *Daqu*, and the remaining central part was named the central part of *Daqu*. The central part of each *Daqu* is about 1.0 kg, and the surface part is 0.7 kg. All samples were made in triplicate. *Daqu* samples were crushed into powder by an antiseptic mortar and stored at −80°C for further experiments.

### Determination of Physicochemical Indices

The content of total acidity was determined according to the method of potentiometric titration. The contents of moisture and reducing sugar were determined according to the atmospheric drying method and 3,5-dinitrosalicylic acid (DNS) method, respectively. The activity of α-amylase was determined according to the starch–iodine method. The activity of acid protease was determined according to the Folin phenol method; one unit of acid protease activity was defined as the amount of tyrosine produced by decomposing casein for 1 min under the conditions of the measurement. The ability of saccharification was determined by measuring released reducing sugars using the DNS method. One unit of saccharification ability was defined as the decomposition of soluble starch to produce 1 mg of glucose per hour under the assay conditions (Li Z.M. et al., 2015; Li et al., 2020). The ability of esterification was determined according to the method of Kumar and Satyanarayana (2009). One unit of esterifying ability was defined as the content of ethyl caproate produced after 100-h reactions.

### Analysis of *Daqu* Microbial Community by a Culture-Dependent Method

#### Determination of Microbial Count

The microbial count was determined according to the method of Du and Xu (2012) and Li Z.M. et al. (2015). The dilution was coated on lysogeny broth (LB) agar medium and cultured at 37°C for 24 h in a thermostatic incubator (FYL-YS-280L, Beijing, China) for the isolation and enumeration of total aerobic bacteria. Similarly, lactic acid bacteria (LAB) were isolated and enumerated on de Man, Rogosa, and Sharp (MRS) agar medium and were cultured at 37°C for 48 h. Yeast and mold were isolated and enumerated on potato dextrose and Rose Bengal Agar medium, respectively, and were cultured at 28°C for 48 h.

#### DNA Extraction and PCR Amplification

Bacterial genomic DNA was isolated using a TIANamp Bacteria DNA Kit (Tiangen, Beijing, China) following the manufacturer’s specifications. The domain of 16S rDNA sequence was amplified using forward primer 27F (5′-AGAGTTTGATCCTGGCTCAG-3′) and reverse primer 1492R (5′-GGTTACCTTGTTACGACTT-3′) (Han et al., 2013). Fungal genomic DNA was extracted by using a plant genomic DNA kit (Tiangen, Beijing, China), according to the manufacturer’s specifications. For the yeast strains, forward primer NL1 (5′-GCATATCAATAAGCGGAGGAAAAG-3′) and reverse primer NL4 (5′-GGTCCGTGTTTCAAGACGG-3′) were used to amplify the D1/D2 domain of the 26S rRNA gene (Wu et al., 2013). For mold, forward primer ITS1 (5′-TCCGTAGGTGAACCTGCGG-3′) and reverse primer ITS4 (5′-TCCTCCGCTTATTGATATGC-3′) were used to amplify the ITS regions (Ng et al., 2012). PCR amplification was conducted with PCR equipment (ABI GeneAmp 9700, United States) and reagents in a 25-μl reaction solution. The final PCR products were analyzed by 0.8% agarose gel electrophoresis and stored at −20°C for further sequencing analysis.

#### Sequencing and Phylogenetic Analysis

The PCR products of 16S rRNA gene of bacteria, 26S rRNA gene of yeast, and ITS gene of molds were sequenced by Shenggong Bio, Shanghai, China. And the manual alignment of the sequences was conducted with DNASTAR software (version 4.0). Sequence homologies were inspected by comparing the acquired DNA sequence with those in the NCBI library^1^.

### Analysis of *Daqu* Microbial Community by Illumina MiSeq Sequencing

We took out one third of each of the three parallel samples to make a mixed sample for Illumina MiSeq Sequencing research. Metagenomic DNA was extracted using a Soil Genomic DNA Purification Kit (Tiangen, Beijing, China) following the manufacturer’s instructions. For bacteria, the 16S rRNA gene was amplified according to the method of Li et al. (2020). The 18S rRNA gene fragments in the fungi were amplified by using the bar-coded primer pairs of SSU0817F (5′-TTAGCATGGAATAATRRAATAGGA-3′) and SSU1196R (5′-TCTGGACCTGGTGAGTTTCC-3′) (Rousk et al., 2010). The PCR amplification program was operated as described by Li L. et al. (2015). The PCR products were sent to Majorbio Bio-Pharm Technology Co., Ltd. (Shanghai, China) and run on an Illumina MiSeq PE300 platform. The raw 16S rRNA and 18S rRNA gene sequencing reads were demultiplexed, quality-filtered by fastp version 0.20.0, and merged by FLASH version 1.2.7 with the following criteria: (i) The 300-bp reads were truncated at any site receiving an average quality score of <20 over a 50-bp sliding window, truncated reads shorter than 50 bp were discarded, and reads containing ambiguous characters were also discarded. (ii) Only overlapping sequences longer than 10 bp were assembled according to their overlapped sequence. The maximum mismatch ratio of the overlap region was 0.2. Reads that could not be assembled were discarded. (iii) Samples were distinguished according to the barcode and primers, and the sequence direction was adjusted, with exact barcode matching and two nucleotide mismatches in primer matching. Operational taxonomic units (OTUs) with 97% similarity cutoff were clustered using UPARSE version 7.1, and chimeric sequences were identified and removed. The taxonomy of each OTU representative sequence was analyzed by RDP Classifier version 2.2 against the 16S rRNA database (Silva v138) using a confidence threshold of 0.7.

### Statistical Analysis

The experiment was performed three times, except for Illumina MiSeq sequencing. And statistical analysis was plotted with Excel 2013 and Origin 8.0 software. And the significant differences (*P* < 0.05) between the means of samples were tested by one-way analysis of variance (ANOVA) using the SPSS 17.0 software (SPSS Inc., Chicago, IL, United States). MEGA 5.0 software was used to construct the phylogenetic tree.

## Results

### The Physicochemical Indices in Different Parts of *Daqu*

As shown in Table 1, the moisture and acidity contents in the central part of *Daqu* were significantly higher (*P* < 0.05) than that of the surface part of *Daqu*, and the content of acidity in the central part was 110% higher than the surface part of *Daqu*. However, the content of reducing sugars, the activity of acid protease and α-amylase, the ability of esterification and saccharification in the central part of *Daqu* were significantly lower (*P* < 0.05) than those of the surface part of *Daqu*. Among them, the content of reducing sugars in the surface part was 146% higher than that of the central part of *Daqu*. The activity of acid protease and α-amylase in the surface part was 171% and 120% higher than that of the central part of *Daqu*, respectively. And the ability of esterification and saccharification in the surface part was 122% and 114% higher than that of the central part of *Daqu*, respectively.

**TABLE 1:** Physicochemical indices of *Daqu* of special-flavor liquor relative.

**Sample**	**Moisture (%)**	**Acidity (mmol/10 g)**	**Reducing sugars (g/100 g)**	**Acid protease activity (U/g)**	**Esterification ability (mg/g)**	**α -Amylase activity (U/g)**	**Saccharification ability (U/g)**
Surface part of *Daqu*	12.06 ± 0.82^a^	1.52 ± 0.12^a^	1.69 ± 0.07^a^	61.43 ± 4.75^a^	49.73 ± 2.74^a^	962.00 ± 10.49^a^	432.11 ± 17.77^a^
Central part of *Daqu*	13.14 ± 0.17^b^	1.67 ± 0.08^b^	1.16 ± 0.1^b^	35.87 ± 2.70^b^	40.89 ± 3.32^b^	804.00 ± 5.42^b^	378.40 ± 31.26^b^

*Values followed by different letters are significantly different for different cultures at *P* < 0.05.*

### Enumeration of Representative Bacterial and Fungal Populations

Table 2 showed the microbial counts of bacteria and fungi in the different parts of *Daqu* samples, including total aerobic bacteria, LAB, yeast, and mold. There was no significant difference (*P* > 0.05) in the total quantities of bacteria between the surface and central parts of *Daqu*. The LAB amounts in the central part of *Daqu* were significantly higher (*P* < 0.05) than those of the surface part of *Daqu*. Compared with yeast and mold, bacteria were the dominant microorganism in *Daqu*. The counts of mold in the central part of *Daqu* were fewer than that of the surface part of *Daqu*. In addition, yeast had low levels in both the surface and central parts of *Daqu*, and yeast was primarily focused on the surface part of *Daqu*.

**TABLE 2:** Microbial count in surface and central parts of *Daqu*.

**Microbial type**	**Microbial counts (CFU/g)**	
	
	**Surface**	**Central**
Total bacteria	2.5 ± 0.3 × 10^9a^	2.4 ± 0.3 × 10^9a^
Lactic acid bacteria	1.6 ± 0.2 × 10^8c^	2.4 ± 0.3 × 10^8b^
Yeast	1.8 ± 0.6 × 10^4d^	1.1 ± 0.1 × 10^2e^
Mold	1.5 ± 0.3 × 10^8c^	1.3 ± 0.1 × 10^8cd^

*Values followed by different letters are significantly different for different cultures at *P* < 0.05.*

### Analysis of Microbial Community by the Culture-Dependent Method

#### Bacterial Community

**FIGURE 2:**
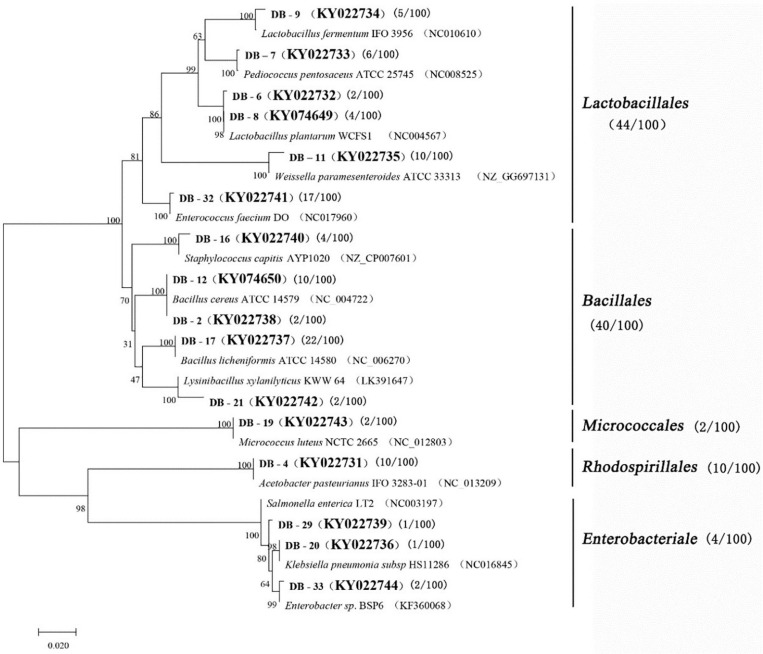
Phylogenetic tree based on l6S rRNA gene sequences.

A total of 100 bacterial strains were randomly selected from the agar plates. Based on the gene sequences of partial 16S rRNAs, the phylogenetic tree showed the remarkable classification among the 14 bacterial species (Table 3 and Figure 2). The bacteria were divided into five categories at the phylotype level, namely, Lactobacillales, Bacillales, Rhodospirillales, Enterobacteriales, and Micrococcales, which accounted for 44%, 40%, 2%, 10%, and 4% of the bacterial community in *Daqu*, respectively. And 94% of bacteria were under the three categories of Lactobacillales, Bacillales, and Rhodospirillales. Bacillales mainly appeared in the central part of *Daqu*, and *Bacillus licheniformis* was the most abundant bacterial community. However, there was no significant difference in the distribution of Lactobacillales in the different parts of *Daqu*. *Enterococcus faecalis* had the largest number of 17 strains in Lactobacillales, while *Lactobacillus plantarum* and *Pediococcus pentosaceus* had six strains. *Acetobacter pasteurianus* with 10 strains belonged to Rhodospirillales and was mainly detected on the surface part of *Daqu*. Enterobacteriales, including *Staphylococcus capitis*, *Klebsiella pneumoniae*, and Enterobacteriales sp., accounted for 4% of the bacteria in *Daqu* and mainly appeared on the surface part of *Daqu.*

**TABLE 3:** Blast and distribution information of bacterial strains of special-flavor *Daqu*.

**Strain**	**Surface (CFU/total CFU)**	**Central (CFU/total CFU)**	**Approximate sequences**	**Similarity rate (%)**	**Species**
DB-17	4	18	NC006270	99	*Bacillus licheniformis*
DB-12	2	10	NC004722	99	*Bacillus cereus*
DB-29	1	0	NC003197	99	*Salmonella enterica*
DB-32	10	7	NC017960	99	*Enterococcus faecalis*
DB-19	1	1	NC012803	99	*Micrococcus luteus*
DB-6/8	2	4	NC004567	99	*Lactobacillus plantarum*
DB-7	3	3	NC008525	99	*Pediococcus pentosaceus*
DB-4	9	1	NC013209	99	*Acetobacter pasteurianus*
DB-9	2	3	NC010610	99	*Lactobacillus fermentum*
DB-11	5	5	NZ697131	99	*Weissella paramesenteroides*
DB-21	0	2	LK391647	99	*Lysinibacillus xylanilyticus*
DB-16	1	3	NZ007601	99	*Staphylococcus capitis*
DB-20	1	0	NC016845	99	*Klebsiella pneumonia*
DB-33	1	1	KF360068	99	*Enterobacter* sp.

#### Fungal Community

**FIGURE 3:**
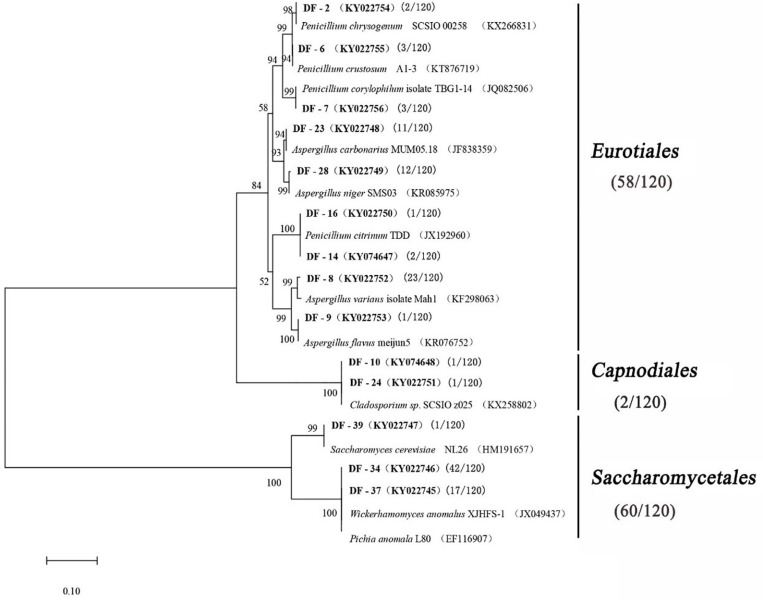
Phylogenetic tree based on the 26S rRNA and ITS gene sequences.

A total of 120 fungal strains were randomly selected, and a phylogenetic tree was constructed for the sequence fragments from the fungus (Table 4 and Figure 3). The fungus was divided into three categories at phylotype level, namely, Saccharomycetales, Eurotiales, and Capnodiales, which had 60, 58, and 2 strains in the *Daqu*, respectively. Saccharomycetales mainly appeared on the surface part of *Daqu*, including *Wickerhamomyces anomalus* (47 strains), *Pichia anomalus* (17 strains), and *Saccharomycetales cerevisiae* (one strain). *Aspergillus* and *Penicillium* belonged to Eurotiales, and *Aspergillus* genus included *Aspergillus varians* (23 strains), *Aspergillus niger* (12 strains), *Aspergillus carbonarius* (11 strains), and *Aspergillus flavus* (one strain). *Penicillium* genus included *Penicillium citrinum* (three strains), *Penicillium chrysogenum* (two strains), *Penicillium crustosum* (three strains), and *Penicillium corylophilum* (three strains).

**TABLE 4:** Blast and distribution information of fungal strains of special-flavor *Daqu*.

**Strain**	**Surface (CFU/total CFU)**	**Central (CFU/total CFU)**	**Approximate sequences**	**Similarity rate (%)**	**Species**
DF-34	34	8	JX049437	99	*Wickerhamomyces anomalus*
DF-37	12	5	EF116907	99	*Pichia anomalus*
DF-14/16	2	1	JX192960	99	*Penicillium citrinum*
DF-8	13	10	KF298063	99	*Aspergillus varians*
DF-28	10	2	KR085975	99	*Aspergillus niger*
DF-23	6	5	JF838359	99	*Aspergillus carbonarius*
DF-9	1	0	KR076752	99	*Aspergillus flavus*
DF-2	1	1	KX266831	99	*Penicillium chrysogenum*
DF-6	2	1	KT876719	99	*Penicillium crustosum*
DF-7	2	1	JQ082506	99	*Penicillium corylophilum*
DF-10/24	2	0	KX258802	99	*Cladosporium* sp.
DF-39	1	0	HM191657	99	*Saccharomyces cerevisiae*
DB-20	1	0	NC016845	99	*Klebsiella pneumonia*
DB-33	1	1	KF360068	99	*Enterobacter* sp.

### Analysis of Microbial Community by Illumina MiSeq Sequencing

Table 5 showed the diversity, richness, and coverage estimations of each data set. The Shannon diversity index, a measurement of overall diversity, indicated the diversity of the microorganisms. Good’s coverage result, an estimator of sampling completeness, highlighted good overall sampling with levels of 99–100%. A total of 93,466 Illumina MiSeq sequencing reads were obtained. Approximately 16,886–24,092 effective reads with different phylogenetic OTUs ranging from 13 to 89 were obtained from the *Daqu* samples for microbial communities. Based on the results of Illumina MiSeq sequencing, the number of bacterial OTUs was more than that of fungal OTUs. Bacterial diversity on the surface part of *Daqu* was lower than that on the central part of *Daqu*, whereas fungal diversity was similar between the surface and central parts of *Daqu*.

**TABLE 5:** Alpha-diversity of special-flavor *Daqu*.

**Type**	**Sample**	**Reads**	**OTU**	**Coverage**	**Abundance index**		**Diversity index**	
						
					**Ace**	**Chao**	**Shannon**	**Simpson**
16S rRNA	Surface	16,886	83	0.99	86	86	1.97	0.3110
	Central	17,853	89	0.99	89	89	2.54	0.1516
18S rRNA	Surface	24,013	13	1	13	13	1.12	0.4701
	Central	24,092	14	1	14	14	1.00	0.4658

**FIGURE 4:**
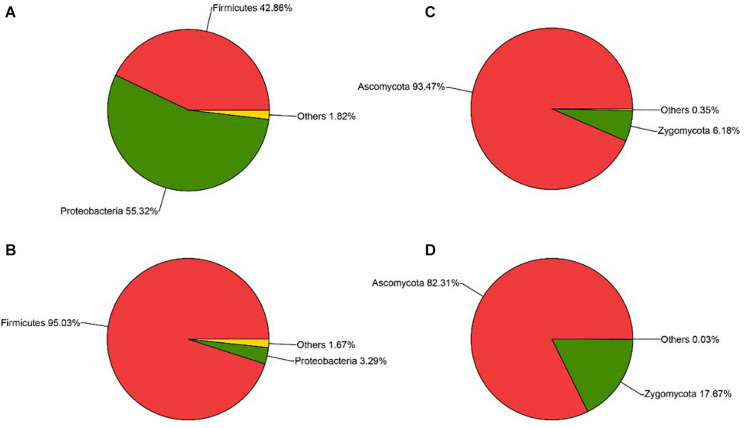
The relative abundances of surface *Daqu* **(A)** and central *Daqu* **(B)** in bacterial communities at the phylum level, and the relative abundances of surface *Daqu* **(C)** and central *Daqu* **(D)** in fungal communities at the phylum level.

**FIGURE 5:**
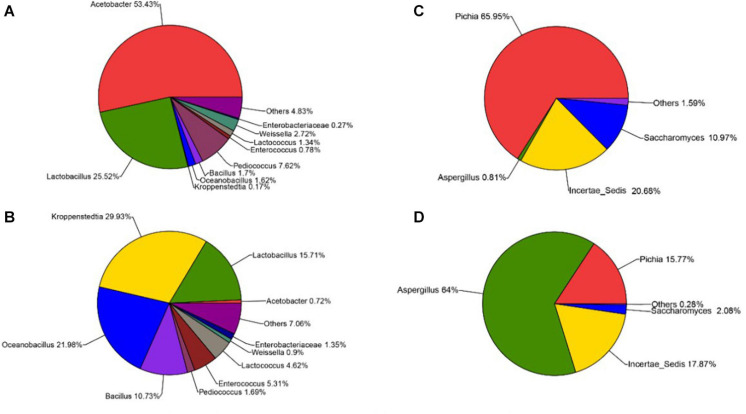
The relative abundances of surface *Daqu* **(A)** and central *Daqu* **(B)** in bacterial communities at the genus level, and the relative abundances of surface *Daqu* **(C)** and central *Daqu* **(D)** in fungal communities at the genus level.

The sequencing results also showed that different parts of *Daqu* had various microbial community structures. For bacterial communities (Figures 4A,B), three phyla were identified on the surface and central parts of *Daqu*. Proteobacteria and Firmicutes were the dominant bacterial communities on the surface part of *Daqu*, which averaged 55.32% and 42.86% of the bacterial communities, respectively. However, bacterial communities on the central part of *Daqu* were dominated by Firmicutes, which averaged 95.03% of the communities. At the genus level (Figures 5A,B), over half of the classified bacteria was *Acetobacter* genus (53.4%), followed by *Lactobacillus* (25.52%) and *Pediococcus* (7.42%) genera in the surface part of *Daqu*. However, *Lactobacillus*, *Kroppenstedtia*, *Oceanobacillus*, and *Bacillus* genera were the abundant strains in the central part of *Daqu*, which accounted for 15.7%, 29.9%, 21.9%, and 10.7%, respectively. Moreover, *Lactococcus*, *Weissella*, *Enterococcus*, and *Enterobacter* genera occurred throughout the surface and central parts of *Daqu*.

Compared with that of bacteria, the fungal community structure was relatively low. At the phylum level, Ascomycota and Zygomycota were detected through sequencing analysis. Ascomycota was the predominant type in both the surface and central parts of *Daqu*, which accounted for 93.47% and 82.31% of the total sequences, respectively (Figures 4C,D). Figures 5C,D showed that *Pichia*, *Aspergillus*, and *Saccharomyces* were the predominant genera. The abundance of *Pichia* and *Saccharomyces* on the surface part of *Daqu* was higher than that on the central part of *Daqu*. However, the abundance of *Aspergillus* genus accounted for 64% of the central part of *Daqu*, but few on the surface part of *Daqu*.

## Discussion

Most of the microorganisms in the Chinese *Baijiu* brewing process come from pit mud, fermented grains, and *Daqu*. Among them, the main source is from *Daqu* (Zou et al., 2018). The traditional process of making *Daqu* is to naturally enrich a variety of microorganisms in *Daqu*. These microorganisms compete to grow in *Daqu* and finally adapt to the changing environment of *Daqu* to survive and reproduce in large numbers. Microbial communities in *Daqu* will also produce rich enzymes and metabolites in their growth and metabolism process and ultimately give the Chinese *Baijiu* a unique flavor (Gan et al., 2019). Some studies have found that *Bacillus* genus could break down starch by producing amylase in *Daqu* and used decomposition products to generate nitrogenous flavor compounds, such as diverse pyrazines (Mukherjee et al., 2009; Zheng et al., 2012). Meantime, the different microbial communities in the different parts of the *Daqu* block led to different physicochemical indices, such as the moisture content, acidity content, and temperature. Similarly, moisture and acidity contents in different parts of *Daqu* were also key environmental factors that could cause different microbial community distributions (Yan et al., 2013). Our study found that moisture and acidity contents in the central part of *Daqu* were significantly higher than those in the surface part of *Daqu*, and the counts of microorganisms were significantly different between the surface and central parts of *Daqu*. Li et al. (2019) also found that the moisture content was significantly different between the surface and central parts of *Yanghe Daqu*, and the different microbial communities were closely related to moisture content in *Daqu*. Therefore, it is of great significance to explore the relationship between the specific species of microbial community and physicochemical indices in *Daqu* for improving the quality and yield of Chinese special-flavor *Baijiu*.

We used culture-dependent and Illumina MiSeq sequencing methods to determine the specific species of the microbial communities in the different parts of special-flavor *Baijiu Daqu*. Our results showed that compared with yeast and mold, bacteria were the dominant microbial community in special-flavor *Baijiu Daqu*. Firmicutes and Proteobacteria were predominant in *Daqu* at the phylum level. Sun et al. (2016) and Gan et al. (2019) also found that Firmicutes and Proteobacteria were the predominant microbial communities in *Jiang*-flavor *Daqu* and *Nong*-flavor *Daqu*. This result indicated that Firmicutes and Proteobacteria may be the main microbial communities in the fermentation process of Chinese *Daqu*, and this also explained why the special-flavor *Baijiu* has the partial flavor of *Jiang*-flavor *Baijiu* and *Nong*-flavor *Baijiu* (Zheng and Han, 2016).

In our study, two bacterial groups, Bacillales and Lactobacillales, were the dominant bacterial communities in special-flavor *Daqu*. However, we found an interesting phenomenon that Bacillales, including the genera of *Bacillus*, *Kroppenstedtia*, and *Oceanobacillus*, were only dominant in the central part of *Daqu*. This was consistent with the results of Illumina MiSeq sequencing. Li et al. (2013) also found that the members of the Bacillales family were dominant in the central part of Fen-*Daqu*. However, the reason for the high number of *Bacillus* genus in the central part of special-flavor *Daqu* needs further study in the future. Jin et al. (2019) found that *Bacillus* genus was the α-amylase producer, which could consecutively transform the raw material of *Daqu* into the key metabolite, such as pyruvate, and lead to further production of organic acid and ethanol in *Jiang*-flavor *Baijiu Daqu*. This may be one of the reasons that the acidity in the central of *Daqu* was significantly higher than that in the surface part of special-flavor *Baijiu Daqu*. We also found that *B. licheniformis* was the most abundant species in Bacillales. This observation was consistent with result of light-flavor *Baijiu Daqu* (Zhang et al., 2014). And Wang P. et al. (2017) found that *B. licheniformis* could not only increase the content of some flavor compounds, such as aromatic substances and pyrazines, but also influence the related fermentation–enzyme activity of *Daqu*, such as increasing the activity of α-amylase. However, we found that the activity of α-amylase in the surface part of *Daqu* was significantly higher than that in the central part of *Daqu*. The reason for this phenomenon may be that the number of fungal communities in the surface part of *Daqu* was higher than that in the central part of *Daqu*, and the ability of some fungal communities to produce the activity of α-amylase was significantly higher than that of *B. licheniformis*, resulting in the different α-amylase activities between the surface and central parts of *Daqu*. α-Amylase is a key enzyme that breaks down the starch of *Daqu* to produce reducing sugar and can provide energy and substrate for the growth of microorganisms and the formation of flavor compounds (Wu et al., 2009). The result of reducing sugar content also indirectly proved this conclusion. We found that the content of reducing sugar in the surface part of *Daqu* was significantly higher than that in the central part.

In our study, Lactobacillales mainly included *Lactobacillus*, *Pediococcus*, *Enterococcus*, *Lactococcus*, and *Weissella* genera, wherein *Lactobacillus* genus was the most abundant bacterial community. Moreover, a considerable number of LAB, including *Enterococcus faecium*, *Pediococcus pentosaceus*, *Lactococcus plantarum*, *Lactococcus fermentum*, and *Weissella paramesenteroides* were identified by the culture-dependent method. These bacterial communities also generally appeared in other Chinese traditional *Daqus* (Sun et al., 2016; Wang X.D. et al., 2017). Zou et al. (2018) found that the members of LAB were the main producers of lactic acid, which is subsequently found in the synthesis of ethyl lactate by esterification in *Nong*-flavor *Daqu*. These microorganisms could survive in high-acid concentration and control spoilage bacteria by secreting bacteriocin. Lactobacillales had multiple metabolic products, wherein lactic acid was its main metabolic product and the main basic substance for the formation of ethyl lactate and other flavor compounds. Moreover, lactic acid could reduce the harsh taste of *Baijiu*, enhance the mellow taste of the liquor body, prolong liquor aftertaste, and strengthen the sweetness of the Chinese *Baijiu* (Wang X.S. et al., 2017). Therefore, these bacterial communities were the crucial microbial communities in the *Daqu*. In addition, our study showed a higher abundance of *Acetobacter* genus in *Daqu*; *Acetobacter* genus occurred in the natural environment of several plants, such as fruits and grains, which could disintegrate the different types of sugars and alcohols to organic acids in oxygen as final substances by a special aerobic metabolism method (Sengun and Karabiyikli, 2011). Sun et al. (2016) found that *Acetobacter* genus play an important role during the brewing process of *Nong*-flavor *Baijiu* and could contribute to the accumulation of ethyl acetate, which has a significant positive effect on the flavor of *Nong*-flavor *Baijiu*. Therefore, *Acetobacter* genus in the *Daqu* may be an important bacterial community that provides the flavor compound during the special-flavor *Baijiu* fermentation process. In the current study, the relative abundance of *Acetobacter* genus was more than 50% in the surface part of *Daqu* but few in the central part of *Daqu*. This result may be due to *Acetobacter* genus being a type of aerobic bacteria. The lack of oxygen led to the reduction of *Acetobacter* genus in the central part of *Daqu*. *Lactobacillus* and *Acetobacter* genera are the main acid-producing bacteria in *Daqu*, and in our study, the abundance in the surface part of special-flavor *Baijiu Daqu* was significantly higher than that in the central part. On the contrary, the content of acidity in the surface part of *Daqu* was significantly lower than that of the central part. In addition to the *Bacillus* genus we mentioned earlier, which was also the main acid-producing bacteria, another reason may be due to the high esterification ability in the surface part of *Daqu*, which resulted in a large amount of organic acids being used as precursors to generate ester compounds (Liu et al., 2017). The result of esterification ability also indirectly proved this conclusion. However, the difference of acid content changes and the accumulation of flavor compounds in different parts of special-flavor *Baijiu Daqu* need further study in the future.

Our study also indicated that the diversity of fungal community was relatively low compared with the bacteria community, which was consistent with the previous studies in other traditional *Daqu* (Fan et al., 2018). The reason for this phenomenon may be that the bacteria were able to survive in harsher environments and exhibited better tolerance compared with fungus under conditions of high moisture content, acidity content, and temperature (Huang et al., 2017). In addition, the counts of yeast had the lowest level in both the surface and central parts of *Daqu*. Among them, *S. cerevisiae* was not detected by Illumina MiSeq sequencing, and only one strain was obtained and isolated by the culture-dependent method. This result was also observed by using the microbial composition analysis of Fen-*Daqu* (Zheng et al., 2012). The reason for this phenomenon may be that *S. cerevisiae* and other yeasts were vulnerable to environmental factors, such as low moisture (below 15%) and high temperature (over 50°C), resulting in the most yeast being killed in the process of medium–high temperature *Daqu* (Li et al., 2013; Gou et al., 2015). Therefore, only few yeasts could survive in the maturation of *Daqu*. In our study, the low moisture content (<13.14%) also proved this conclusion and may be the reason for this result. Wang and Xu (2015) found that the number of molds in *Daqu* was higher than that of yeast, because they had relatively high tolerance to low moisture content and could form spores to resist higher temperatures. However, the acidity content in the central part of *Daqu* was significantly higher than that in the surface part of *Daqu*, which was not suitable for the growth of molds, resulting in the number of molds in the central part of *Daqu* being lower than that in the surface part. Saccharomycetales and Eurotiales dominated the fungal communities in our study. The culture-dependent method was used to determine fungal communities which were composed of the *Wickerhamomyces*, *Pichia*, and *Aspergillus* genera. Likewise, the *Pichia* and *Aspergillus* genera were detected with a relative abundance of more than 50% in the surface and central parts of *Daqu* by Illumina MiSeq sequencing, respectively. Some studies have indicated that the *Wickerhamomyces* and *Pichia* genera were ester-producing yeasts with the high ability of saccharification and esterification. *Pichia* could generate the etherification of various alcohols, such as ethanol, geraniol, isoamyl alcohol, and 2-phenylethanol, resulting in increasing concentrations of esters with a fruity aroma, because alcohols and acids are key substrates for ester synthesis (You et al., 2016). Our study also found that the abundance of *Wickerhamomyces* and *Pichia* genera on the surface part of special-flavor *Baijiu Daqu* was significantly higher than that on the central part. At the same time, the results of saccharification and esterification ability in the surface part of special-flavor *Baijiu Daqu* were significantly higher than those of the central part also prove that they were undoubtedly important functional fungal communities in special-flavor *Daqu*.

Our study also found that *Aspergillus* was predominant in special-flavor *Daqu*, including *A. varians*, *A. niger*, and *A. carbonarius*, which were also consistent with a previous study on the fungal community of *Maotai Daqu* (Wang et al., 2008). Esterification ability is an important indicator of the ability in *Daqu* to produce flavor compounds. It refers to the ability of acid to combine with alcohol to remove one water molecule to form an ester (Kumar and Satyanarayana, 2009). Meantime, acid protease can decompose the protein in *Daqu* raw materials into small-molecule substances (polypeptides, free amino acids, and so on), which has a positive effect on the formation and quality of Chinese *Baijiu* flavor (Huang et al., 2017). Hu et al. (2017) found that a considerable number of *Aspergillus* genus could produce high esterification ability and acid protease activity, which is of great significance for improving the quality of *Jiang*-flavor *Daqu*. However, there was a significant difference in the distribution of *Aspergillus* genus between the surface and central parts of *Daqu*. The *Aspergillus* genus was mainly located in the central part of *Daqu*. However, our research found that the acid protease activity and esterification ability in the central part of *Daqu* were significantly lower than that in the surface part of *Daqu*. This may be because the partial metabolic mechanism of *Aspergillus* genus was inhibited in a highly acidic environment, resulting in the decreasing acid protease activity and esterification ability. However, the specific mechanism behind the decrease of acid protease activity and esterification ability needs further study. In other words, these results showed that the microbial community had a unique distribution in special-flavor *Baijiu Daqu*. Meantime, the distribution of the unique microbial community in special-flavor *Baijiu Daqu* also had a significant impact on the enzyme activity in different parts of *Daqu*.

Other bacteria, including *K. pneumoniae*, *Salmonella enterica*, and *Micrococcus luteus*, had low number in both the surface and central parts of *Daqu*. Tang et al. (2019) found that the rich LAB in the *Xiaoqu* could inhibit the growth of these bacteria by producing large amounts of lactic acid. Similarly, some fungus also was found in our study, such as *Penicillium* genus. They were common in traditional Chinese *Daqu*s, and the *Penicillium* genus would reduce *Baijiu* yield and exhibit adverse effects on *Baijiu* quality (bitter taste of base *Baijiu*) (Zheng et al., 2011; Yan et al., 2013). Of course, these bacteria also were discovered in *Daqu* samples maybe because of external pollution. Li et al. (2013) found that these microorganisms could be killed during the distillation process and did not survive in the *Baijiu* with high alcohol concentration. Therefore, more information on the microbial community needs to be studied during the special-flavor *Baijiu* fermentation process.

All in all, the dominant microbial communities in different parts of *Daqu* had been detected by culture-dependent and Illumina MiSeq sequencing methods, such as *Lactobacillus*, *Bacillus*, *Weissella*, *Pediococcus*, and *Acetobacter* genera. However, the results obtained by different methods were slightly different, and culture-dependent and Illumina MiSeq sequencing methods still have their own advantages and disadvantages (Wang X.D. et al., 2017; Yang F. et al., 2019). The culture-dependent method cultivates the microorganisms in *Daqu* through different selective media to obtain pure strains and then amplifies and sequences specific fragments. The sequence information obtained is complete, and a large amount of information is carried in the fragments, so that each strain information can be observed more intuitively and quickly. However, the disadvantage is that the workload is large, only cultivable microorganisms can be identified, and the obtained *Daqu* microbial diversity is incomplete (Zheng et al., 2012). In our study, *Kroppenstedtia* and *Oceanobacillus* genera were not detected by the culture-dependent method. In contrast, we had obtained the existence of these microbial communities through the Illumina MiSeq sequencing method in *Daqu*, and they have a higher abundance compared to other microbial communities. Our study indicated that the results obtained on microbial community through the Illumina MiSeq sequencing method are more comprehensive; we have identified 54 genera and can determine the distribution and relative abundance of various genera in *Daqu*, thereby further determining the dominant microbial community in different parts of *Daqu*. The Illumina MiSeq sequencing method also has the advantages of being a simple and fast operation (Sun et al., 2016). However, its disadvantage is that it is easily restricted by various factors, such as genome extraction, sequence depth, and algorithms. Meantime, compared with the culture-dependent method, the results of Illumina MiSeq sequencing did not detect *Penicillium* genus, possibly due to its very low content or insufficient sequence information, which could not be classified. These results showed that we could effectively explain the microbial community changes in Chinese *Baijiu* fermentation industry based on the advantages of culture-dependent and Illumina MiSeq sequencing methods.

## Conclusion

In this study, we used culture-dependent and Illumina MiSeq sequencing methods to analyze the microbial structure of special-flavor *Daqu*. We found that bacterial diversity was higher than fungal diversity in *Daqu* and that there were significant differences in microbial composition, distribution, and physicochemical indices between the surface and central parts of *Daqu*. These results could provide a certain reference for the production and utilization of special-flavor *Daqu* in the future. However, more information on the function microbial community in special-flavor *Baijiu Daqu* need to be studied for a better understanding of the relation between microbial community and physicochemical indices.

## Data Availability Statement

The original contributions presented in the study are publicly available. This data can be found here: NCBI, accession numbers and links in Supplementary Table 1.

## Author Contributions

YC and KL participated in all the experimental processes and wrote the initial manuscript. TL and RL made the figures and tables. YW proofread the revised manuscripts. GF and FZ provided the design and financial support of this study. All authors contributed to the article and approved the submitted version.

## Conflict of Interest

The authors declare that the research was conducted in the absence of any commercial or financial relationships that could be construed as a potential conflict of interest.

## Footnotes

1 http://www.ncbi.nlm.nih.gov/
