# Supplementary material for: Analysis of Difference in Microbial Community and Physicochemical Indices Between Surface and Central Parts of Chinese Special-Flavor Baijiu Daqu
Source: Front Microbiol. 2021 Jan 14;11:592421. doi: 10.3389/fmicb.2020.592421 (PMC7840566; doi:10.3389/fmicb.2020.592421)
Supplement: Supplementary file 1 [file Table_1.DOCX]

**The specific link of microbial sequencing data in the Genbank of NCBI library**

*Lactobacillus fermentum* strain DQ-4 16S rRNA gene, partial sequence. <https://www.ncbi.nlm.nih.gov/nuccore/KY022734.1/>

*Pediococcus pentosaceus* strain DQ-3 16S rRNA gene, partial sequence. <https://www.ncbi.nlm.nih.gov/nuccore/KY022733>

*Lactobacillus plantarum* strain DQ-2 16S rRNA gene, partial sequence. <https://www.ncbi.nlm.nih.gov/nuccore/KY022732>

*Lactobacillus plantarum* strain DQ-15 16S rRNA gene, partial sequence. <https://www.ncbi.nlm.nih.gov/nuccore/KY074649>

*Weissella paramesenteroides* strain DQ-5 16S rRNA gene, partial sequence. <https://www.ncbi.nlm.nih.gov/nuccore/KY022735>

*Enterococcus faecium* strain DQ-11 16S rRNA gene, partial sequence. <https://www.ncbi.nlm.nih.gov/nuccore/KY022741>

*Staphylococcus capitis* strain DQ-10 16S rRNA gene, partial sequence. <https://www.ncbi.nlm.nih.gov/nuccore/KY022740>

*Bacillus cereus s*train DQ-16 16S rRNA gene, partial sequence. <https://www.ncbi.nlm.nih.gov/nuccore/KY074650>

*Bacillus cereus* strain DQ-8 16S rRNA gene, partial sequence. <https://www.ncbi.nlm.nih.gov/nuccore/KY022738>

*Bacillus licheniformis* strain DQ-7 16S rRNA gene, partial sequence. <https://www.ncbi.nlm.nih.gov/nuccore/KY022737>

*Lysinibacillus xylanilyticus* strain DQ-12 16S rRNA gene, partial sequence. <https://www.ncbi.nlm.nih.gov/nuccore/KY022742>

*Micrococcus luteus* strain DQ-13 16S rRNA gene, partial sequence. <https://www.ncbi.nlm.nih.gov/nuccore/KY022743>

*Acetobacter pasteurianus* strain DQ-1 16S rRNA gene, partial sequence. <https://www.ncbi.nlm.nih.gov/nuccore/KY022731>

*Salmonella enterica* strain DQ-9 16S rRNA gene, partial sequence. <https://www.ncbi.nlm.nih.gov/nuccore/KY022739>

*Klebsiella pneumoniae* strain DQ-6 16S rRNA gene, partial sequence. <https://www.ncbi.nlm.nih.gov/nuccore/KY022736>

*Enterobacter* sp. strain DQ-14 16S rRNA gene, partial sequence. <https://www.ncbi.nlm.nih.gov/nuccore/KY022744>

*Penicillium chrysogenum* strain DQ-29 rRNA gene, partial sequence. <https://www.ncbi.nlm.nih.gov/nuccore/KY022754>

*Penicillium crustosum* strain DQ-30 rRNA gene, partial sequence. <https://www.ncbi.nlm.nih.gov/nuccore/KY022755>

*Penicillium corylophilum* strain DQ-31 rRNA gene, partial sequence. <https://www.ncbi.nlm.nih.gov/nuccore/KY022756>

*Aspergillus carbonarius* strain DQ-23 rRNA gene, partial sequence. <https://www.ncbi.nlm.nih.gov/nuccore/KY022748>

*Aspergillus niger* strain DQ-24 rRNA gene, partial sequence. <https://www.ncbi.nlm.nih.gov/nuccore/KY022749>

*Penicillium citrinum* strain DQ-25 rRNA gene, partial sequence. <https://www.ncbi.nlm.nih.gov/nuccore/KY022750>

*Penicillium citrinum* strain DQ-32 rRNA gene, partial sequence. <https://www.ncbi.nlm.nih.gov/nuccore/KY074647>

*Aspergillus varians* strain DQ-27 [rRNA gene, partial sequence. https://www.ncbi.nlm.nih.gov/nuccore/KY022752](%20rRNA%20gene,%20partial%20sequence.%20https://www.ncbi.nlm.nih.gov/nuccore/KY022752)

*Aspergillus flavus* strain DQ-28 rRNA gene, partial sequence. <https://www.ncbi.nlm.nih.gov/nuccore/KY022753>

*Cladosporium* sp. strain DQ-33 rRNA gene, partial sequence. <https://www.ncbi.nlm.nih.gov/nuccore/KY074648>

*Cladosporium* sp. strain DQ-26 rRNA gene, partial sequence. <https://www.ncbi.nlm.nih.gov/nuccore/KY022751>

*Saccharomyces cerevisiae* strain DQ-22 26S rRNA gene, partial sequence. <https://www.ncbi.nlm.nih.gov/nuccore/KY022747>

*Wickerhamomyces anomalus* strain DQ-21 26S rRNA gene, partial sequence. <https://www.ncbi.nlm.nih.gov/nuccore/KY022746>

*Pichia anomalus* strain DQ-20 26S rRNA gene, partial sequence. <https://www.ncbi.nlm.nih.gov/nuccore/KY022745>
